# 

**Published:** 1847-06

**Authors:** 


### INDEX TO VOLUME THIRD.

Page.

Annalist, N. Y. 59, 384
Alum in Pertussis, 238
Apothecaries, 240, 572
Anatomy, legalization of, 243
"Athletæ," 312
Aneurisms, observations on, by O. Bryan Bellingham, 321
Apoplexy, Cases of, 456
Aborigines, physical condition of, 462
Academy of Medicine, N. Y. 398
Anatomist, pocket, 510
Acne Rosacea, case of, 598
Asclepias Tuberosa, by T. T. Lockwood, M D. 600
Address, New-Year's, 635
Atelectasis, 665
Barrett's Moses Address, 60
Blaakman's Dr. Address, 60
Braithwait's Retrospect. 61
Bulwer Sir E. L. on Hydropathy, 186
Beck on the effect of blisters, 212
Beck on blood letting, 552
Baldwin on poisonous properties of Quinia, 271
Bladder, chronic inflammation of, treated by injections Nitrate of Silver, by Dr. McDonnell, 286
Brook's letters on veternary medicine, 300
Beef tea, 425
Blake James, M. D. on action of medicine 468, 609
Breast, abcess of, 488
Blooodletting in the young subject, 542
Brainard D. M. D., on astringent and stimulating injections in suppuration, 618
Brainard on cure of Spina Bifida, 685
Bigelow S. L. on Ethereal Solution of gun cotton for wounds, 695
Colegrove B. H., M. D. on Empyema, 6
Convention National Medical, Proceedings of, 31, 175
Convention National Medical, reports of Committees, 40, 97
Colegrove E. on lectures of Sir A. Cooper. 93
Correspondent Foreign, letter from, 199
College of Physicians and Surgeons, N. Y. 246
Concours, Election by, 309
Calculus Urinary, by G. D. Stevens, M. D. 333
Cutaneous eruptions induced by medicines, 356
Children, food of, 357
Clinical instruction, remarks on, 366
Condie on diseases of children, 382
Chelius' Surgery, by South, 394, 527, 602
Constipation, fatal case of, 416
Conjunctivitis, exterior use of nitrate of silver in, 419
Cholera, progress of in Europe and Asia, 425
Carpenter's human physiology, 441

Page.

Coventry, Prof. C. B. on Stomatitis Materna, 513
Croup, application of nitrate of silver to larynx, 545
Chloroform, 561, 573, 672, 702, 710
Duodenum, diseases of, by S. Salisbury, M. D 1
Dunglison's charge to graduates, 59
Dispensary Medical, Buffalo College, 89
Dunglison Prof. 244
Disinfecting fluid, Burnett's, 360
Dowler, Bennett, M. D. criticisms and controversies relating to nervous and muscular systems, 378
Dictionary, medical, by Gardner, 511
Drugs, spurious, 568
Dieffenback, memoirs of, 621
Dropsy after Scarlatina, warm bath for, 694
Empyema, case and successful operation, by B. H. Colegrove, M. D. 6
Ether Sulphuric, inhalation of, 121
Ether Sulphuric in reduction of dislocations, 152
Ether Sulphuric in labour, by Walter Channing, M. D. 254, 280
Ethics, Code of, adopted by National Convention, 203
Ethics, Remarks upon, 239
Erie Co. Med. Society, 149
Erysipelas Epidemic, by H. Jewett, M. D. 257
Epilepsy, by Editor of Southern Medical Journal, 355
Epilepsy by Rostan, Velpeau, Williams, &c. 415
Enteritis follicular, by Editor, 385, 449
Errors, typographical etc. 703
EnteritisGastro follicular, Endemic, 733
Fever Typhus at New York, 59
Fever, Ship, 120, 165, 225, 293, 342, 687
Fever, Yellow at New Orleans, 487, 699
Freeman Wm. case of, 350
"Firing," treatment of Paralysis by, 413
Flagg on treatment of young permanent teeth, 422
Fractures, mode of treatment by Jobert, 616
Fever, treatment of, by A. G. Henry, M. D. 627
Green Horace, M. D. on diseases of the air passages. Review, 125
Griffith's Medical Botany, 183
Genital organs of female, diseases of 478
Griscom's report of New York Hospital, 537
Generation, tracts on, 699
Gun cotton ethereal solution of, 694, 701
Green Caleb, M. D. on chloroform, 710
Ganson H., M. D. case of cervical tumour and operation, 714
Harvard University, new Professors in, 56
Hamilton's Prof. Surgical Dispensary, 65, 660, 730
Hamilton on enlarged tonsils, 190
Hamilton on ovarian hernia, 334

Page.
Hyatt's anatomical preparations, 123
Hydrocephalus, Iodide of Potassum in, 187
Herrrick on Med. Dept. of Army, 537
Homœopathy, 361, 421
Hahnemanns, character and writings, by editor of the New York Jour. of Medicine, 371
Herrick on surgery at Buena Vista, 413
Heart, extraction of needle from, 418
Halloway's pills, 421
Hopkins Rev. Asa T. Report of illness, death and autopsy, by Prof. Hamilton, 460
Health public. 756
Hiccough, treatment of, 494
Hospital Buffalo city, 502
Hospital N. Y., Griscoms report of, 537
Hallowell Edward, M. D. on Endemic gastro-follicular enteritis, 733
Insanity, curability of, by Pliny Earle, M. D. 548
Influenza, 704
Jackson's catalogue of Anat. museum, 765
Jones on Ophthamic Medicine and Surgery, 179
Jewett H., M. D. on Epidemic Erysipilas, 258
Jarvis' Surgical Adjuster, 760
Knapp's M. L. Address, 60
Kreosote, a remedy for vomiting, 247, 416
Kellogg A. O., M. D. on Acne Rosacea, 598
Latham on Heart. Review. 872
Latham on subjects connected with clinical medicine, 380
La Ford Corydon M. D. resolutions complimentary to, 55
Lee C. A. Prof. Address, 61
Lee C. A. Catalogue Med. Plants, 758
Lockwood T. T., M. D. on term of utero gestation in man and inferior animals, 95
Lockwood on Eupatoreum perfoliatum, 197
Lockwood on Asclepias Tuberosa 600
Lawrence on the Eye, 125
Liver, removal of! 499
Lectures Introductory, 577, 641, 715
Liston Robert, memoir of, 621
Lectures Medical, plan to insure attendance upon, 634
Motts' Velpeau's Surgery, 58
McClellan Dr. George, Obituary, 68
Medical Colleges, statistics of, 54
Medical College of Buffalo, 496, 573, 763
Malpractice, trial for, and one thousand dollars damages, 145
Malpractice, suits for, 196, 220, 447
Mendenhall's Vade Mecum 182
Medicines, adulteration of, 314
Meningitis Cerebro-spinal Epidemic in Missouri, 358
Medulla spinalis, effusion of serum in theca, 420
Murphy on Parturition, 506

Page.
Microscopes, American, 509
Medical College at Columbus, 511, 701
Meachem T. G., M. D. on penetrating wounds of thorax, 705
Neil on Veins and Lymphatics, 382
Oedema of Glottis, Surgical treatment of, 173
Opium in inflammation, 228
Ophthalmic memoranda, 640
Oleum Tiglii, over dose of, 912
Parker Prof. Clinique, 691
Peyer's glands in Fever, 174
Pseudo-doctors, 245
Prescriptions, per centage on, 313, 575
Physicians of Paris, sketches of, 404
Palmer's artificial limbs, 492
Physicians female, 492
Paine's Martyn Prof. Materia Medica, 637
Pride J. B., M. D. on Oleum Tiglii, 712
Peaslee's Synopsis, 763
Quarantine hospital N. Y. 186
Quack advertisement, 320
Revere Prof. Obituary, 61
Ranking's Abstract, 351, 704
Rostan on cerebral affections, 409
Roux on luxation of elbow, 411
Reid W. W., M, D. on apoplexy, 500
Rush Medical College 687
S. Salisbury, M. D. on diseases of Duodenum, 1
Strychnia in Intermittent fever by D. Brainard M. D. 159
Spinal column, distortions of, 233
Stephens G. D., M. D. on Urinary Calculus, 332
Strychnia, death by, 361
Strong Woodbridge, M. D. on Typhus, 426
Strawberry leaf, 491
Strychnia taken by mistake for Morphia, 512
Stomatitis Materna 513
Society of the State of New York, officers of, 636
Stille's General Pathology, 725
Turner on Young Physic, 148
Tongue, Semeiology of, 231
Tucker's Midwifery, 759
University of New York Medical Department, appointments in, 248
University of Pennsylvania, Medical Department, Report of Faculty, 305
Vogel's Pathological Anatomy, 123
Valerianate of Zinc, 365
Vaccine Virus origin of, 678
Warren Prof. Valedictory, 59
White Prof. James P. Compliment to, 122
Wilson's Human Anatomy, 123
Wood's Practice of Medicine, 255
Wood's Quarterly Retrospect, 383
Watson's Principles and practice of Physic, 446
Ware on Croup, 555
Yandell T. W., on Medical matters. and Medical men in Paris, 300

*Statistics of Medical Schools.*

The following list embraces all the schools of which we have been able to obtain reports for the present year:—

| | CLASS. | GRADUATES. |
|---|---|---|
| University of Pennsylvania, | 411 | |
| Jefferson Medical College, | 493 | 170 |
| Pennsylvania Medical College, | 95 | 33 |
| Franklin Medical College, | | 5 |
| University of state of New-York, (College Physicians and Surgeons), | 194 | 50 |
| University of city of New-York, | 410 | 123 |
| Massachusetts Medical College, | 165 | 34 |
| Medical Department Yale College, | | 28 |

| | | |
|---|---|---|
| Medical Dept. Transylvania University, | 205 | 64 |
| Medical University of Louisville, | 354 | 75 |
| Medical College of Ohio, | 170 | 53 |
| Willoughby Medical College. | 101 | 38 |
| Cleveland Medical College, | 206 | |
| Memphis Medical College, | 55 | |
| Castleton Vermont Medical,College, | 131 | 42 |
| University of Missouri, (St. Louis), | | 28 |
| University of St. Louis, | | 13 |
| Albany Medical College. | 100 | 30 |
| Rush Medical College, | 70 | 17 |
| Indiana Medical College, | | 19 |
| Medical College of Georgia, | 106 | 33 |
| Medical Dept. University of Buffalo, | 68 | |
| Geneva Med. College, | 180 | 43 |
| Medical College S. Carolina, | 203 | 74 |
| Richmond Va. Medical College, | 84 | 21 |

---

*To Readers and Correspondents.*

Subscribers are requested to delay having the second volume bound until they receive an index, which will be forwarded with the next number.

The Publishers desire to state that the inducements offered to delinquent subscribers to remit the amount of subscription due, can only be extended to those who remit WITHOUT DELAY.

---

*Errata.*—A portion of this number having gone to press in the absence of the Editor, several literal errors, which would be likely to escape notice of a proof-reader who was not the writer of the communications, will be found to have occurred, especially in the first portion of the *review of Dr. Latham's work.* Our readers will greatly oblige us if they will make on the margin the following corrections, some of which are very important to the sense:—

Page 8th, 23d line, for *cardic*, read *cardiac.* Page 8th, 26th line, for *tittle*, read *title.* Page 8th, 28th line, for *one*, read *oral.* Page 9th, 13th line, for *accumen*, read *acumen.* Page 9th, 19th line, for *preceding*, read *subseqnent.* Page 9th, 33d line, for *to be pursued*, read *difficult to be pursued.* Page 9th, 26th line, for *cordial*, read *cardiac.* Page 10th, 7th line, for *Practioner*, read *Practitioner.* Page 10th, 12th line, for *worthy*, read *vastly.* Page 11th, 15th line, for *anœmia*, read *anœmic.* Page 12th, 3d line, for *percussion*, read *maximum.* Page 13th, 22d line, for *been*, read *seen.* Page 13th, 22d line, for *writes*, read *writers.* Page 13th, 25th line, for *stethescope*, read *stethoscope.* Page 14th, 39th line, for *Gothic* (!), read *Gallic.* Page 14th, 39th line, for *and*, read *in.* Page 15th, 4th line, for *directions*, read *dissections.* Page 15th, 15th line, for *a desire*, read *an occasion.* Page 15th, 16th line, for *firmness*, read *fairness.*

